# Assessment of novel probiotic strains on growth, hematobiochemical parameters, and production costs of commercial broilers in Bangladesh

**DOI:** 10.14202/vetworld.2021.97-103

**Published:** 2021-01-13

**Authors:** S. M. Tanjil Shah, Md. Tanvir Islam, Rakiba Zabin, Pravas Chandra Roy, Nigar Sultana Meghla, Iqbal Kabir Jahid

**Affiliations:** Department of Microbiology, Jashore University of Science and Technology, Jashore 7408, Bangladesh

**Keywords:** Cobb 500 broiler, *Enterococcus faecium*, *Pediococcus acidilactici*, probiotics, serum biochemistry

## Abstract

**Background and Aim:**

The use of antibiotic growth promoters (AGPs) in the poultry industry has raised concern because of their potential harm to human health. Emerging evidence suggests that probiotics are a safer substitute, although little research has explored this in Bangladesh. We recently isolated local bacterial strains with probiotic properties. We aimed to determine their impact on the growth, hematobiochemical parameters, and production costs of broiler chicks relative to that of a commercial probiotic (CP) and AGP.

**Materials and Methods:**

Day-old male broiler chicks (Cobb 500, n=63) were divided equally into three experimental groups (three replicates per group and seven chicks per replicate). First group was fed a basal diet supplemented with the AGP, ciprofloxacin (CTL group), second group was fed a basal diet supplemented with the CP, Protexin^®^ (CP group), and the third group was fed a basal diet supplemented with our isolated bacterial strains (study probiotic [SP] group) for 36 days. Body weight was recorded daily, and relative growth rate (RGR), feed conversion ratio (FCR), and organ weights and carcass yields were calculated at the study’s end. Blood obtained on day 36 was used to determine the number of red blood cell (RBC) and white blood cells (WBCs), hemoglobin (Hb) concentration, packed cell volume, erythrocyte sedimentation rate, as well as levels of serum glucose, total and high-density lipoprotein (HDL) cholesterol, and triglycerides (TGs). Total production costs were estimated by summing the variable and fixed costs.

**Results:**

Chicks in both the CP and SP groups experienced significant decreases in blood glucose levels and significant increases in BW, RGR, FCR, levels of RBC and WBC, Hb concentration, and packed cell volume compared with those in the CTL group (p*<*0.05 for all). Our data suggested a numerical reduction (p>0.05) in levels of total cholesterol, TGs, and HDL in the SP and CP groups when compared to the CTL group. In addition, both CP and SP treatments resulted in significant (p<0.05) gains in net profit compared with the treatment given to the CTL group.

**Conclusion:**

Administration of probiotics, either from a commercialized or local source, led to greater improvements in growth, hematological parameters, and net profits of broiler chicks when compared with that of an AGP. This suggests that they are suitable alternatives to the AGPs used in poultry feed and that our isolated strains, in particular, are an ideal option for farmers in Bangladesh.

## Introduction

Recently, poultry has become one of the ­highest consumed animal proteins worldwide due to its affordable price, nutrient density, and compatibility with many religious dietary laws. Catering to this demand, many farmers have begun incorporating antibiotic growth promoters (AGPs) into their animal feed as a means to ensure a good feed conversion rate, inhibit pathogenic bacteria, and reduce rates of mortality [1]. Contemporary data indicate, however, that this may pose a threat to human health as residues from AGPs in poultry have been found to alter their intestinal microbiota and lead to the generation of antibiotic-resistant bacteria. Out of concern for long-term consequences, scientific committees in several countries are prohibiting the use of AGPs that do not serve therapeutic purposes [2,3]. Unfortunately, such restrictions have increased the incidence of poultry diseases and resulted in economic losses. To solve this problem, research efforts should focus on isolating and identifying suitable microorganisms, namely, probiotics that can support poultry development. According to the International Scientific Association for Probiotics and Prebiotics, “when administered in adequate amounts, probiotics confer health benefits to the host” [4]. In addition, investigations show that probiotics contribute to the maintenance of healthy gut flora, boost feed intake and conversion, improve digestion, and protect from pathogens [5]. Probiotics’ diverse roles in suppressing cancer, lowering serum glucose, and lowering serum cholesterol [6,7] are leading to increased acceptance of probiotics.

To the best of our knowledge, few studies in Bangladesh have evaluated the effects of commercial probiotic (CP), AGPs, or enzyme supplements on poultry growth [8,9]. Notably, when Hasan *et al*. [10] assessed hematobiochemical parameters in broiler chicks following intake of the probiotic, Protexin^®^ (Novartis Bangladesh Ltd.), the enzyme, Alquerzim^®^ (ACI Ltd., Bangladesh), or the liver tonic, Livavit^®^ (Square Pharmaceuticals Ltd., Bangladesh), findings revealed that the probiotic had a negligible impact. Indeed, the viability of commercially available probiotics may be far less than what their product labels actually claim [9], which is why our team has endeavored to characterize the bacteria from yogurt and goat milk samples sold in Bangladesh. Previously, we isolated and identified the strains of *Enterococcu*s *faecium* and *Pediococcus acidilactici* and reported that they possessed antagonistic activity, tolerance to phenols, bile salts, and NaCl, were capable of surviving in simulated gastric juice (+/− lysozyme) and adhering to ileum epithelial cells, as well as passed the milk coagulation, hemolytic assay, and antibiotic susceptibility tests [11].

For the current study, we explored how the aforementioned isolated probiotics affect growth, hematobiochemical profiles, and production costs of broiler chicks against a CP and AGP.

## Materials and Methods

### Ethical approval

The experiment was permitted by the Ethical Review Committee, Faculty of Biological Sciences, Jashore University of Science and Technology, Jashore, Bangladesh (certification number: ERC/FBST/JUST/2019–32). Every effort was made to lessen the pain and harms to experimental animals.

### Study period and location

The study was conducted from March 2019 to May 2019. All experiments were performed at the Department of Microbiology, Jahsore University of Science and Technology, Jahsore, Bangladesh. Experimental birds were reared in a poultry farm near University campus.

### Experimental groups

Day-old male broiler chicks (Cobb 500, n=63) were purchased from Nourish Poultry and Hatchery Ltd. and divided equally into three groups (three replicates per group and seven chicks per replicate). A floor litter system was used to keep the chicks in separate pens, and all chicks became acclimated to the experimental conditions over 2 days.

To assess the efficacy of the probiotics, each group of chicks was fed a different diet for 36 days. As local poultry farms in Jashore, Bangladesh, usually rear birds for 36 days, we conducted our study for 36 days. In particular, the control (CTL] group was fed a basal diet (Nourish Poultry Feed Ltd., Bangladesh) supplemented with the AGP, Ciprofloxacin (Renaflox^®^ powder, Renata Ltd., Bangladesh, Supplementary Table-1). The CP group was fed a basal diet supplemented with the multi-strain probiotic, Protexin^®^ (Probiotic International Ltd., UK, Supplementary Table-2). The study probiotic mixture (SP) group was fed a basal diet supplemented with a mixture of two strains of *E. faecium* and three strains of *P. acidilactici* (Table-1), which our group had isolated from yogurt except for that of *P. acidilactici B.1*, being isolated from Black Bengal goat’s milk instead [11]. We note that AGPs were not given to the latter groups receiving probiotics, and that the CP and SP groups, were administered 1 g Protexin^®^/L drinking water (according to the manufacturers’ recommendations) or 2×10^8^ colony-forming unit/100 mL drinking water respectively.

**Table-1:** Composition of study probiotic mixture (SP).

Bacteria	CFU/100 mL
*Enterococcus faecium 14/1*	4×10^7^
*Enterococcus faecium 12/1*	4×10^7^
*Pediococcus acidilactici 12/3*	4×10^7^
*Pediococcus acidilactici B.1*	4×10^7^
*Pediococcus acidilactici 13/1*	4×10^7^

### Animal management and growth assessment

The temperature in the facility where the chicks were housed was maintained at 35°C during the 1^st^ week and between 27°C and 29°C for the remainder of the study. Vaccinations against Newcastle disease (on 5^th^ and 24^th^ day), infectious bursal disease (on 10^th^ and 20^th^ day), and hydropericardium syndrome (on 17^th^ day) were administered to birds. Fresh drinking water was provided *ad libitum*. During the 36-day fattening period, feed consumption per day was determined by subtracting the amount of food remaining in the chicks’ pens from the specified quantity they received the day prior. Feed consumption per chick was determined by dividing the total amount of feed consumed by the total number of chicks in each group. Feed conversion ratio (FCR) was calculated each day according to the method described by Wagner *et al*. [12]. Body weight (BW) was measured in triplicate and recorded before the experiments each day, as well as analyzed for weight gain every 4^th^ day. Relative growth rate (RGR) was calculated using the formula described by Crampton and Lloyd [13].

### Determination of organ weights and carcass yields

Two chicks from each replicate were randomly selected to be weighed, numbered, and slaughtered on day 36 of the study. Following these procedures, chicks were defeathered and had their heads, necks, shanks, feet, and viscera carefully removed. The resulting carcasses were then dressed and weighed with the dressing percentage estimated according to the procedure by Brake *et al*. [14]. Finally, the liver, spleen, gizzard, and heart were each weighed and their percent contribution to overall BW was calculated by totaling their weights and expressing this sum as a percentage of live BW.

### Evaluation of hematological parameters

Blood was drawn from the wing veins of two randomly selected chicks of each replicate on day 36 of the study. Complete blood counts, lipid profile and hemato-biochemical parameters were determined. Approximately 4 mL of blood samples from each sacrificed bird was collected from the jugular vein into plain tubes (for biochemical analysis) and anticoagulant tubes (for hematological analysis). Hematological analysis was conducted using automatic SYSAM-XN-1000, XN-550 AL Random Access Hematology Machine (SYSMEX CORPORATION, Japan) and the biochemical analysis was conducted by Siemens Dimension RxL/Max/Vitros350 Random Access Chemistry Analyzer (Siemens Healthcare Diagnostics Inc, USA) after obtaining the serum through centrifugation. Next, the number of erythrocytes (red blood cell [RBC]) and leukocytes (white blood cell [WBC]) was counted and expressed, in million/cumm and thousand/cumm of blood, respectively. Concentration of hemoglobin (Hb) was estimated and expressed as g/dL, packed cell volume was determined using the Wintrobe hematocrit tube, and erythrocyte sedimentation rate (ESR) was obtained using the equation set forth by Lamberg and Rothstein [15].

PCV (%) = Red blood cells’ height (cm)/Height of Total blood’s height (cm) × 100.

### Evaluation of biochemical parameters

Blood samples were collected from two randomly selected chicks of each replicate on day 36 of the study and incubated in test tubes overnight at 4°C. Samples were then centrifuged at 3000 rpm for 10 min to obtain serum, which was kept frozen until further analysis. Levels of total cholesterol, high-density lipoprotein (HDL) cholesterol (HDL), triglycerides (TGs), and blood glucose were estimated by enzymatic assays using Randox lipid and glucose reagents (Randox Laboratories Ltd., UK), and a photoelectric colorimeter (Model: AP-101, Japan**)** [16].

### Cost–benefit analysis

Variable costs pertaining to the feed, chicks, AGP, and production costs [17], fixed costs regarding building rent, equipment, management of chicks (i.e., labor, vaccines, disinfectants, and husbandry supervision), and uncertain costs for the fluctuations in the currency value and price of the dead chicks were all summed to yield the total estimated cost. In specific, the price of each chick was Tk 48, feed price was Tk 41.55 per kg during the experimental period, and the fixed cost was Tk 50 per chick. On day 36 of the study, total returns in money were calculated by multiplying the live BW of each chick by their price per kg [17], and then, net profit was calculated by subtracting total costs from total returns [18].

### Statistical analysis

Analysis of variance was conducted to assess group differences in growth, and hematological and biochemical parameters. When differences were significant, Duncan’s multiple range test was applied to measure specific differences between pairs of means. All data were analyzed using SAS software 9.1 (SAS Inst. Inc., NC, USA) and presented in graphs using GraphPad Prism 8.0 (GraphPad Software, USA).

## Results and Discussion

We assessed the efficacy of locally isolated bacterial strains as probiotics on growth, hematological and biochemical parameters, and cost savings in broiler chicks against that of the CP, Protexin^®^, and an AGP. Throughout the study period, initial BW, weight gain, and FCR were comparable across all treatment groups.

### Growth

#### Weight gain

**Figure-1:**
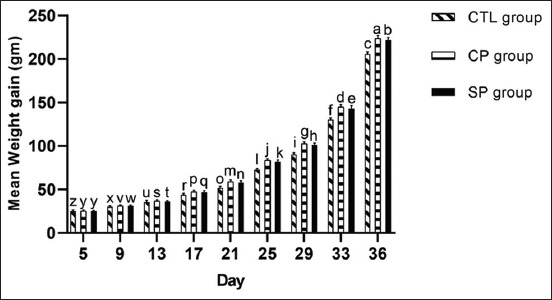
Weight gain (g) of broilers fed basal diets supplemented with antibiotic (CTL group), commercial probiotic Protexin^®^ (CP group), and study probiotic mixture (SP group). Values are mean body weight gain at every 4^th^ days’ interval. Within each day, different letters indicate significant difference in values at p*<*0.05 (ANOVA, Duncan’s MRT).

On day 1, the average BW was 52.75 g and no statistical differences in initial BW were detected between the three groups. After 36 days of treatment, however, mean BW of the CTL, CP, and SP groups was 2293.75 g, 2533.75 g, and 2503.00 g, respectively. From this, analyses discovered that BW had increased significantly within the CP and SP groups by the study’s end (p<0.05, Figure-1). These results echo those of the previous studies demonstrating that broilers fed *Bacillus*-based multi-strain probiotics [19] and birds given *E*. *faecium* [20] gain more weight than their control counterparts. Furthermore, we observed significantly higher weight gain (p<0.05) in both the SP and CP groups like other researchers [21]. Figure-1 demonstrates that maximum WG was obtained at 36 days. During the entire fattening period, overall WG was higher in the SP and CP groups, relative to the CTL group.

#### RGR

**Figure-2:**
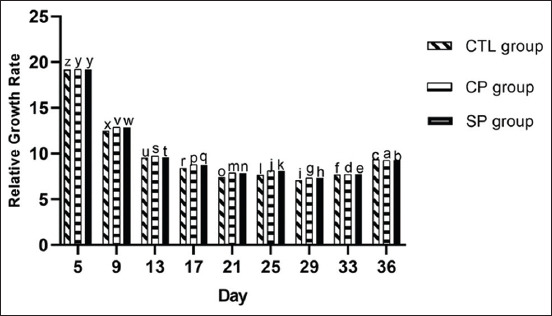
Relative growth rate of broilers fed basal diets supplemented with antibiotic (CTL group), commercial probiotic Protexin^®^ (CP group), and study probiotic mixture (SP group). Values are the RGR at every 4^th^ days’ interval. Within each day, different letters indicate significant difference in values at p*<*0.05 (ANOVA, Duncan’s MRT).

For all groups, RGR was 19 on day 5, and then it gradually decreased from day 6 to 21 before increasing to 9.39, 9.25, and 9.28 by day 36 for CTL, CP, and SP group, respectively. Interestingly throughout the entire study, RGR was significantly higher among the CP and SP groups relative to that of the CTL group (p<0.05), and chicks in the CP group maintained the highest RGR (Figure-2). A positive relationship was observed between RGR and daily weight gain in the CP and SP groups (Figures-1 and 2).

#### FCR

**Figure-3:**
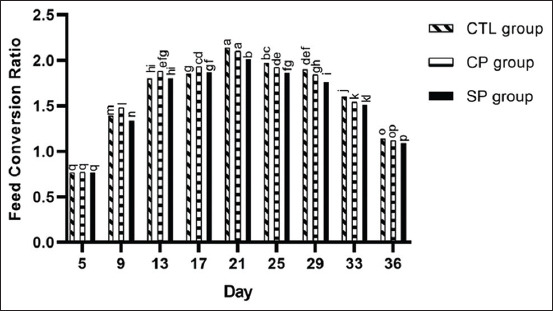
Feed conversion ratio of broilers fed basal diets supplemented with antibiotics (CTL group), commercial probiotic Protexin^®^ (CP group), and study probiotic mixture (SP group). Values are the FCR at every 4^th^ days’ interval. Within each day, different letters indicate significant difference in values at p*<*0.05 (ANOVA, Duncan’s MRT).

We measured feed consumption and estimated FCR on every 4^th^ day interval and observed that three different treatments had significant effects on FCR on different days (Figure-3). From day 1 to 5, FCR was equal across the treatment groups. From day 6 to 21, FCR increased gradually for all chicks, yet values did not differ significantly between the groups on any particular day. From day 21 to 36, FCR decreased gradually with a comparatively lower value in both the SP and CP groups (Figure-3). Such observations align with findings from one related investigation showing that a diet supplemented with *Lactobacillus* does not improve FCR beyond 21 days [22], and another reporting that “providing *Bacillus amyloliquefaciens* as probiotics had a negative linear effect on FCR from day 0 to 35” [23]. However, they also disagree with data from Bai *et al*. [24] demonstrating that antibiotics and probiotics decrease feed/gain ratio during the first 3 weeks of treatment. This incremental increases in FCR through day 21 could be attributed to higher feed consumption and lower daily weight gain, and alternatively, gradual decreases in FCR past day 21 are due to less feed intake and expedited weight gain (Figures-1 and 3).

### Organ and carcass weights

No significant differences with respect to the percentages of the mean organ and carcass weights relative to chicks’ BW were observed in any of the experimental groups (Table-2), which is similar to the results of other studies [25,26]. In addition, chicks in the CP group had the greatest carcass percentage (75.21%), whereas chicks in the CTL group had the lowest carcass percentage (72.69%). Overall, the CP was most effective at enhancing growth followed by our isolated bacterial strains and the AGP. It should be noted that our probiotic mixture contained only two bacterial species, whereas Protexin^®^ contained seven bacterial species and two yeast species. Thus, it is presumable that such outcomes are owed to the larger number of strains in the CP. Along this vein, Hussein and Selim [27] showed that a mixture of multiple probiotics and yeast augmented growth and carcass yield more than that of a single probiotic.

**Table-2:** Effects of dietary probiotic treatments on internal organs weight relative to body weights of broiler chickens (percentages).

	Dietary treatment groups^1^		

	CTL group	CP group	SP group
Carcass	72.69±1.27	75.21±0.89	74.53±1.84
Liver	2.74±1.01	2.63±0.05	2.73±0.30
Heart	0.48±0.03	0.54±0.07	0.48±0.05
Gizzard	1.13±0.34	1.24±0.18	1.27±0.13
Spleen	0.06±0.02	0.07±0.00	0.08±0.01

1 Abbreviation: CTL=Control, CP=Commercial probiotic; SP=Study probiotic mixture. The data are presented as Mean±SD

### Hematological parameters

A significant increase in production of RBCs, WBCs, and Hb concentration was observed in the CP and SP groups relative to the CTL group (p<0.05, Table-3), conforming to results reported by other researchers [28-30]. But, we did not observe any differences in ESR among the three groups. However, we observed a higher count in RBC for the CP (3.55±0.02 million/cumm) and SP (3.35±0.04 ­million/cumm) group compared with the CTL group (3±0.07 million/cumm). Similarly, for Hb concentration, we observed a higher concentration in both the CP (12.2±0.6 g/dL) and SP (11±0.61 g/dL) fed groups compared to that of the CTL group (9.6±0.41 g/dL). Furthermore, WBC count was also significantly enhanced in the CP group (20.705±0.007 thousand/cumm) and SP group (18.64±0.04 thousand/cumm) relative to the CTL group (14.835±0.025 thousand/cumm) (p<0.05). Collectively, these findings suggest that probiotics help bolster immunity in chicks.

**Table-3:** Effects of dietary probiotic treatments on hematological parameters of broiler chickens.

Hematological parameters	Dietary treatment groups^1^		

	CTL group	CP group	SP group
RBC (million/cumm)	3^c^±0.07	3.55^a^±0.02	3.35^b^±0.04
WBC (thousand/cumm)	14.835^c^±0.025	20.705^a^±0.007	18.64^b^±0.04
Hb (g/dL)	9.6^c^±0.41	12.2^a^±0.6	11^b^±0.61
PCV (%)	24.5^c^±0.07	31.5^a^±0.26	28^b^±0.59
ESR (mm)	3±0	2±0	2.7±0.42

1 Abbreviation: CTL=Control, CP=Commercial probiotic, SP=Study probiotic mixture. The data are presented as Mean±SD. Within a row, different superscripts indicate a significant difference in values at p<0.05 (ANOVA, Duncan’s MRT)

### Biochemical parameters

We observed minor depletions in levels of total cholesterol, HDL, and TG, although these changes were not significant between the groups (Table-4). Concerning cholesterol, these results partially agree with those noted by Li *et al*. [31] and Ahmadi [32], who saw robust reductions in levels of plasma cholesterol among Shaoxing ducks fed *B*. *subtilis* and blood cholesterol of broilers fed the yeast probiotic, Thepax, respectively. With regard to TG, amounts in the CP and SP groups were slightly below those in the CTL group. This is similar to findings from several lines of research showing larger decreases in serum TG levels in broilers fed CPs over antibiotics [7,21,30]. Finally, both probiotic treatments significantly lowered plasma glucose levels compared to treatment by AGP (p<0.05), which may be unique to our study since Nosrati *et al*. [30] detected no significant changes in serum glucose concentration between antibiotic- and probiotic-treated broilers.

**Table-4:** Effects of dietary probiotic treatments on biochemical parameter of broiler chickens.

Biochemical parameter (mmol/L)	Dietary treatment groups^1^		

	CTL group	CP group	SP group
Cholesterol	3.57±0.31	3.5±0.21	3.3±0.24
TG	0.805±0.2	0.72±0.4	0.735±0.2
HDL	2.3±0.42	2.2±0.28	2.1±0.14
Glucose	12.1^a^±0.5	10.76^b^±0.45	11.1^a,b^±0.45

1 Abbreviation: CTL=Control; CP=Commercial probiotic; SP=Study probiotic mixture,The data are presented as Mean±SD.Within a row, different superscripts indicate a significant difference in values at p<0.05 (ANOVA, Duncan’s MRT)

### Cost–benefit analysis

Analyses revealed that the CP group had the lowest total production costs (330.56 Tk per chick) and the SP group had the highest total production costs (335.09 Tk per chick), although these differences were statistically insignificant (Table-5). It is possible that such increases in production costs are attributed to the heightened feed intake by chicks given probiotics. In addition, net profit per chick was higher in both the CP (Tk 49.13 per chick) and SP (Tk 40.36 per chick) groups compared to that of the CTL group (Tk 11.47 per chick), thereby evidencing that all of the probiotics administered here were more economically efficient than the AGP (p<0.05).

**Table-5:** Cost-benefit analysis of dietary probiotic treatments.

	Dietary treatment groups^1^		

	CTL group	CP group	SP group
Variables costs			
Chick purpose cost (Tk/Chick)	48	48	48
Antibiotic and probiotic cost (Tk/Chick)	70	53	65
Feed intake (Kg/Chick)	3.96	4.32	4.14
Feed cost (Tk/Chick)	164.59	179.56	172.09
Fixed costs			
Labor, vaccines, disinfectants, building rent, etc. (Tk/Chick)	50	50	50
Total production costs (Tk/Chick)	332.59	330.56	335.09
Income			
Mean final weight (Kg/Chick)	2.29	2.53	2.5
Sale price of broiler (150 Tk/kg Chick)	344.06	379.69	375.45
Net profit (Tk/Chick)	11.47^c^	49.13^a^	40.36^b^

1 Abbreviation: CTL=Control; CP=Commercial probiotic; SP=Study probiotic mixture. Within a row, different superscripts indicate a significant difference in values at p<0.05 (ANOVA, Duncan’s MRT)

## Conclusion

In this study, we compared the influence of a mixture of five bacterial strains with probiotic properties, CP, and AGP on the growth, hematological and biochemical parameters, and production costs of broiler chicks. Of these treatments, the probiotics had the most positive impact on outcome measures. This suggests that they are suitable alternatives to AGPs used in poultry feed and that our locally isolated strains, in particular, are an ideal option for farmers in Bangladesh since CP tend to be imported from other countries. Future investigations examining how different combinations of our locally isolated bacteria with prebiotics and/or yeast moderate the chicks’ development will provide further insight into their overall efficacy.

## Authors’ Contributions

SMTS, MTI, and IKJ designed the study. RZ and NSM reared broilers and performed experiments on those. PCR conducted biochemical analyses. SMTS, MTI, and RZ analyzed the data and drafted the manuscript. NSM, PCR, and IKJ revised and finalized the manuscript. All authors have read and approved the final manuscript.

## Supplementary Tables

**Supplementary Table-1:** Composition of the basal diets: starter (d 0–20), grower (d 21–34).

Ingredient	Starter (%)	Grower (%)
Corn, yellow	56.12	60.80
Soybean meal	37.50	32.61
Poultry fat	3.00	3.43
Dicalcium-phosphate	1.75	1.56
Limestone	0.80	0.78
Salt	0.30	0.32
Vitamin premix	0.25	0.25
Dl-Methionine	0.20	0.17
Trace mineral premix	0.08	0.08

Source: Nourish Poultry Feed Ltd., Bangladesh

**Supplementary Table-2:** Composition of probiotic Protexin^®^.

Bacteria	CFU/g
*Lactobacillus plantarum*	1.26×10^8^
*Lactobacillubulgaricus*	2.06×10^8^
*Lactobacillus acidophilus*	2.06×10^8^
*Lactobacillus casei*	2.06×10^8^
*Bifidobacteriumbifidum*	2.00×10^8^
*Streptococcus thermophilus*	4.10×10^8^
*Streptococcus faecium*	5.40×10^7^
*Aspergillusoryzae*	5.32×10^7^
*Torulopsis spp.*	5.32×10^7^

Source: Novartis Limited, India
